# A New Research Model for Artificial Intelligence–Based Well-Being Chatbot Engagement: Survey Study

**DOI:** 10.2196/59908

**Published:** 2024-11-11

**Authors:** Yanrong Yang, Jorge Tavares, Tiago Oliveira

**Affiliations:** 1 NOVA Information Management School (NOVA IMS) Universidade Nova de Lisboa Lisboa Portugal

**Keywords:** artificial intelligence–based chatbot, AI-based chatbot, mental well-being, intention to engage, engagement behavior, theoretical models, mobile phone

## Abstract

**Background:**

Artificial intelligence (AI)–based chatbots have emerged as potential tools to assist individuals in reducing anxiety and supporting well-being.

**Objective:**

This study aimed to identify the factors that impact individuals’ intention to engage and their engagement behavior with AI-based well-being chatbots by using a novel research model to enhance service levels, thereby improving user experience and mental health intervention effectiveness.

**Methods:**

We conducted a web-based questionnaire survey of adult users of well-being chatbots in China via social media. Our survey collected demographic data, as well as a range of measures to assess relevant theoretical factors. Finally, 256 valid responses were obtained. The newly applied model was validated through the partial least squares structural equation modeling approach.

**Results:**

The model explained 62.8% (*R*^2^) of the variance in intention to engage and 74% (*R*^2^) of the variance in engagement behavior. Affect (β=.201; *P=*.002), social factors (β=.184; *P=*.007), and compatibility (β=.149; *P=*.03) were statistically significant for the intention to engage. Habit (β=.154; *P=*.01), trust (β=.253; *P<*.001), and intention to engage (β=.464; *P<*.001) were statistically significant for engagement behavior.

**Conclusions:**

The new extended model provides a theoretical basis for studying users’ AI-based chatbot engagement behavior. This study highlights practical points for developers of AI-based well-being chatbots. It also highlights the importance of AI-based well-being chatbots to create an emotional connection with the users.

## Introduction

### Overview

According to the World Health Organization (2019), >80% of people worldwide face challenges in accessing mental health services [1]. This lack of access can be attributed to various factors, such as inadequate attention to health care, limited availability of medical resources, and the inability to afford the high costs of treatment [1,2]. Accessibility and scalability of mental health services need to be addressed [3].

An artificial intelligence (AI)–based well-being chatbot can engage in conversations with humans in a relatively natural manner, offering companionship, emotional support, and guidance for emotional well-being [4]. Therapeutic well-being chatbots work by simulating how a mental health professional would treat a user [5], and companionship well-being chatbots facilitate or develop a social relationship with the user through chatting to alleviate and channel negative emotions, such as loneliness and irritability [6,7]. These new digital interventions provide considerable relief to individuals who need psychotherapeutic help but are plagued by a lack of time, space, or resources to access it [8-10]. Individuals who have interacted with these chatbots have expressed satisfaction with their experiences and have shown a positive attitude toward the future development of this technology [11]. These chatbots allow users to discuss private topics anonymously, effectively avoiding any feelings of shyness that may arise [12]. Well-being chatbots have also been used by professionals as an effective complementary tool to traditional face-to-face therapy [13,14]. In addition, they contribute positively to the dissemination of mental health knowledge and the promotion of healthy behaviors [11,15].

A growing number of research findings support the idea that digital mental health interventions, for instance, well-being chatbots, reduce the risk of chronic diseases by improving patients’ psychosocial well-being and promoting other health behaviors [16-18]. They can help users overcome barriers to mental health support, and users can anonymously accept help from chatbots [19-21]. Scholars have taken notice of this phenomenon, and chatbot effectiveness, software design and development, use, and user satisfaction are being emphasized [22,23]. However, the problem of low engagement and high dropout rates between users and chatbots have not been prioritized, particularly in studying engagement behaviors through theoretical models. This will severely influence the user experience and effectiveness [3,8]. Exploring the factors influencing users’ engagement behavior with well-being chatbots is critical to comprehend and refine this association, to serve users better [8,24].

This study aimed to investigate user intention to engage with well-being chatbots and engagement behavior by developing a new theoretical model that combines the theory of interpersonal behavior (TIB), diffusion of innovation (DOI), and trust. The goal is to understand the relationships among various factors and analyze their impact on the intention to engage and engagement behavior. We gathered data through a web-based survey to examine this model and identify the relationships between different factors. This research contributes to expanding the existing knowledge on theoretical models, particularly in the context of a human-centered digital mental health intervention. In addition, it will assist in designing, developing, and improving user-centered well-being chatbots; alleviating the problem of mental health medical resources; and helping to improve the overall well-being of the population. We have two research questions related to the objective of this study: (1) What factors influence users engaged with AI-based well-being chatbots? (2) How could AI-based well-being chatbot service be improved using the results of this study to improve users’ engagement and experience?

### Theoretical Background Rationale

Published studies about adoption of AI-based well-being chatbots tend to focus on either emotional or technical components of this technology but not on a more integrated approach to study this new technology [25-30]. Particularly in digital health adoption, the most used theories, the technology acceptance model and unified theory of acceptance and use of technology, mostly focus on general technology adoption drivers [31,32]. Explaining the interaction of AI-based well-being chatbots with users goes beyond a simple technical interaction, it has been documented that they can create a psychological connection, like a friendship [33,34]. Therefore, we use the TIB, specifically its affect construct, to understand the relationship between a user and an AI-based well-being chatbot [29,30]. AI-based well-being chatbots are innovative technologies in the field of mental health care and personal well-being, and the application of DOI theory is beneficial for studying the factors that contribute to the adoption of AI-based well-being chatbots [35]. Trust is a key factor, particularly when dealing with personal and sensitive data [36,37], like the sharing process between the user and AI-based chatbots when it concerns mental health and personal well-being [38]. Without trust in the treatment intervention, the expected health outcomes between both parties may not be achieved [39]. The study brings these theories together through a new approach that combines relevant phycological factors for the adoption of AI-based well-being chatbots, which can be measured with the TIB and trust theory and the technical and innovation component of this new technology, which can be measured with the DOI theory.

### Engagement Behavior in Digital Mental Health Intervention

Mental well-being is an increasingly important health topic of public concern. AI-based chatbots empower mental health and well-being through AI technology to provide emotional support to human beings [40]. AI-based chatbots enable user interaction based on text or voice support and complete corresponding tasks, recognizing users’ emotions and providing solutions [41]. The services of AI-based chatbots for mental well-being as a new digital mental health intervention to users are evolving, and it is crucial to study users’ engagement behavior.

Engagement is a multidimensional concept that includes not only the formation of interest or adherence to a predefined plan, but also the development of trust, integration, and ongoing participation [42]. In this study, engagement behaviors are defined as the behaviors of users interacting with a well-being chatbot. The well-being chatbot serves as a new type of digital health intervention that provides users with mental health self-management and psychotherapy services [42]. Users’ engagement is an important factor, influencing the effectiveness of mental health interventions [43]. Research has shown that high engagement is associated with high intervention effectiveness [29,30,44,45]. In mental health treatment, participation in ongoing treatment is necessary for recovery [46,47]. A study of a digital mental health intervention found that >70% of users failed to complete all treatment modules and >50% withdrew before completing all treatment modules in general [29,48]. An analysis of mental health applications use showed that the average 15-day retention rate was only 3.9% [49]. Another study showed that mobile apps that emphasized user participation in design increased the effectiveness of interventions for depression and anxiety [50]. In a meta-analysis study of the impact of digital mental health engagement on mental health outcomes, users with higher levels of access showed substantial or moderate improvements in postintervention mental health outcomes [29]. This study explores the relationship between factors around engagement behaviors. This will help to uncover the insight of users’ willingness to engage and their engagement behaviors and improve the design capabilities and services of well-being chatbot.

### TIB Theory

TIB was developed by Triandis [51] in 1977. It is similar to the ABC (attitude-behavior-context) model by Stern [52], combining internal and external factors, including affect, social factors, perceived consequences, habit, and facilitating conditions, to understand intended behavior [53]. In the context of engagement with well-being chatbot research, TIB is a well-suited theoretical model because well-being chatbots operate in a way similar to social software, where communication with users is accomplished through text dialogue and voice dialogue [54]. Users communicate trial experiences and results, and even recommend an AI-based chatbot to others [55]. The affect factor can seriously impact an individual’s willingness to communicate [56]. People will recommend their favorite products to each other, and this recommendation behavior will influence the individual’s intention [57]. Individuals past communication habit of using mobile apps will influence their willingness to use them [58]. If individuals frequently use instant messaging apps, they will be accustomed to this online communication method. TIB contains the above 3 critical aspects known as affect, habit, and social factors. Therefore, TIB is chosen as a theoretical basis for our model.

### DOI Theory

DOI describes the process by which people embrace new ideas, use new products, and engage in new practices [59]. In general, only a few people have an attitude of developmental acceptance of new ideas and are willing to try them out and embrace them in the initial stages. As these people propagate them, gradually, more people begin to embrace them; the innovative idea or product thus diffuses through the population and eventually reaches saturation [60].

DOI was proposed by Rogers [59] in 1995, it helps us to understand the characteristics of an innovation and what attracts users to it. According to Rogers’ research, 5 key features influence the adoption of an innovation: relative advantages, complexity, compatibility, observability, and trialability. Well-being chatbots are an innovative technology that has emerged in recent years, but they are not widespread in daily life. As their contribution to the mental health field, studying their dissemination among people leads to its understanding and acceptance by more people can contribute to human health and well-being. Therefore, extending the TIB model by adopting the properties of DOI is crucial. Among the 5 characteristics, observability can be considered equivalent to the combined effect of demonstrability and visibility [61]. Visibility was not used in this study because AI-based chatbot engagement was treated as a personal experience. Still, results demonstrability was used in our research model. Trialability was also not adopted because there was no evidence of whether the user had trialed a chatbot.

### Trust

Trust is defined as the willingness of one party to accept the actions of another party, irrespective of the latter’s ability to control them [62]. The trust placed in machines is determined as the willingness of users to accept the information generated by machines and to adhere to their recommendations [63]. This indicates that one party intents to form a relatively secure attachment to the other, despite the potential for negative outcomes [64]. This represents a psychological mechanism that can reduce uncertainty and increase the likelihood of successful interaction with other entities within the environment [65]. Trust is a prerequisite for any social interaction and is instrumental in fostering collaboration and cooperation between individuals [66]. It serves as a key factor in successful transactions and establishing long-term relationships [67]. In the field of mental health, trust is of paramount importance in the relationship between patients and health care professionals [68,69]. The interactive behavior of users with chatbots for health purposes is analogous to that of patients with their doctors; the establishment of collaborative and cooperative relationships based on trust is conducive to the achievement of health objectives [70,71]. Therefore, trust represents a crucial element in the investigation of engagement with chatbots.

### Research Model and Hypotheses

**Figure 1:**
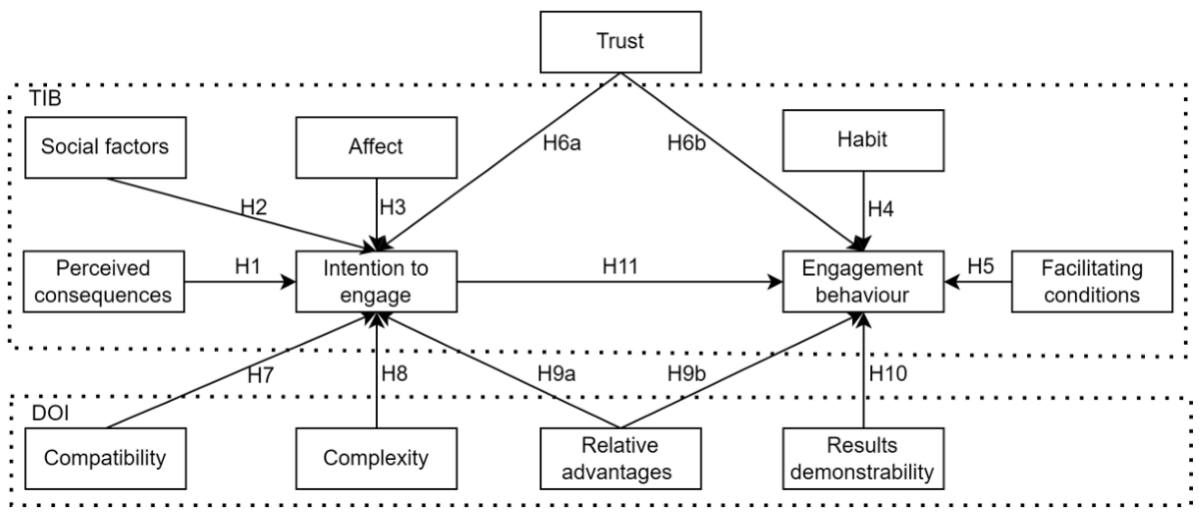
Research model. DOI: diffusion of innovation; H: hypothesis; TIB: theory of interpersonal behavior.

Following the theoretical rationale, TIB, DOI, and trust were combined to support the understanding of users’ intention to engage with AI-based well-being chatbots and engagement behavior in our research, as shown in Figure 1.

### Development of Hypotheses

Perceived consequences are the positive or negative results of an action after it has occurred and the possibility of the outcome occurring [72]. When the perceived consequences are positive, the individual will be prompted to engage with the behavior to achieve advantages; when the perceived consequences are negative, the individual’s willingness to engage with the behavior will be reduced [53]. It had been validated to have a relevant effect on behavioral intention [53,73]. Well-being chatbots offer mental companionship and emotional support, contributing positively to users’ emotional and mental well-being [9]. Therefore, we assume that perceived consequences will be positively related to the intention to engage with well-being chatbots (hypothesis 1).

Social factors are related to the extent to which people are influenced by others who are significant to them [51,73]. Individuals in a group or those observed by a group will comply with some of the unwritten rules within the group, and the likelihood that an individual will act in accordance with the group’s demands increases under the pressure of the group [51,74,75]. Social factors have been shown in several studies to positively influence individual behavioral intention [73,76-78]. In health care, the influence of social factors was examined and affirmed from multiple perspectives in a study of clinicians’ adoption of mHealth (mobile health) tools [79]. In chatbots that provide services in a social context, users decisions are influenced by perceptions of how those around them use these services [78]. Regarding the context of a well-being chatbot engagement, we hypothesize that social factors will be positively related to the intention to engage with well-being chatbots (hypothesis 2).

Affect is used to describe the mental representation of internal bodily sensations associated with emotions, behaviors, or personality tendencies [80]. It is the purely emotional part of an individual’s attitude and contains positive or negative emotions, for instance excitement, joy, depression, and displeasure [81,82]. Affect has been shown to have an influence on behavioral intention in studies on information technology applications [76,83,84]. In the context of AI-based well-being chatbot engagement research, we assume that affect will positively influence individuals’ intention to engage with AI-based well-being chatbots. Thus, we propose that affect will be positively related to the intention to engage with well-being chatbots (hypothesis 3).

Habit is a learned behavior, an automatic response to a steady stream of contextual cues [85], and it is regarded as a major influence on behavior [86]. A study has shown that an individual’s habits can predict future behavior to some extent [87]. Because the popularity of the internet as well as smartphones and the effectiveness of using digital interventions for health behaviors have been proven [51,53,54], this has caused health care apps to gradually become a way to optimize people’s daily health care behavioral habits [88]. As the well-being chatbot serves as a health care information system, we assume that habit will be positively related to engagement behavior (hypothesis 4).

“Facilitating conditions” is a term that refers to objective elements in the environment that enable the easy execution of behavior [51]. In the IT context, it is defined as the resources necessary to support the use of a system, such as access to the internet or a smartphone [31]. Facilitating conditions have been identified as a key factor which influenced individuals’ behavior related to engagement [83]. Thus, we hypothesize that facilitating conditions will be positively related to engagement behavior (hypothesis 5).

Trust has been recognized as one of the critical factors in human-robot interaction research [89,90]. Users’ trust in AI-based chatbots is based on the AI-based chatbot’s performance and services being dependable, trustworthy, and being able to assist in achieving the user’s intended purpose [37]. Developing and nurturing trust in the psychotherapy process to establish a good therapeutic relationship through engagement and ultimately effective treatment is crucial [91,92]. Meanwhile, trust was identified to have a major influence on the intention to act on eHealth websites [93]. Accordingly, trust influences users’ willingness to intent and engage with AI-based well-being chatbots [91-93]. So, we assume that trust positively influences an individual’s intention to engage AI-based well-being chatbots (hypothesis 6a) and trust positively influences users’ engagement behavior (hypothesis 6b).

Compatibility refers to the extent to which the innovation matches the existing values and beliefs, previous experiences, and demands of potential users [59,94]. It provides a good indicator of how extensively an innovation complies with potential users’ lifestyles, needs, and preferences [60]. In previous research, compatibility was identified as one that influenced the intention to behavior [95]. Well-being chatbots meet the real-time needs of users [96-98], and chatbot mobile apps match the habits of smartphone users [99]. In this research, we assume that compatibility will be positively related to the intention to engage with AI-based well-being chatbots (hypothesis 7).

Complexity is a measure of how difficult it is to understand and use an innovation [59]. It is a systematic form that is associated with almost all aspects of health care [100]. Complexity has been proven to have an impact on digital technology in health and well-being apps [101]. In another study on health care chatbots, complexity had a strong impact on the ability of chatbots to successfully provide health information and adoption behavior [100,102,103]. In this research, we assume that low complexity will be positively related to the intention to engage with well-being chatbots (hypothesis 8).

Relative advantages is a term that refers to the degree to which an innovation is better than the object it replaces [94]. Innovation with greater relative advantage is beneficial for its diffusion [104]. It has been shown that an innovation will not be used if potential users believe that there is no comparative advantage in the adoption of the innovation over its earlier counterparts [105]. AI-based well-being chatbots are more empathetic than their earlier counterparts and even have memory functions, these advantages motivate users to interact and engage with them more [96,106-110]. Thus, we assume that relative advantages will be positively related to the intention to engage with AI-based well-being chatbots (hypothesis 9a) and relative advantages will be positively related to engagement behavior (hypothesis 9b).

Results demonstrability is the degree to which innovative results are presented and disseminated [61]. Innovations will be more adopted if they generate demonstrably positive results; if the converse is the case, the chances of the innovation being adopted become lower [94]. Studies have shown that results demonstrability is a potential predictor of behavioral adoption [111]. AI-based well-being chatbots can serve users as an mHealth app. Thus, we assume that results demonstrability will be positively related to the intention to engage with AI-based well-being chatbot engagement (hypothesis 10).

Intention to engage in a behavior is the most direct determinant of an individual’s behavior [112]. Exploring the relationship between intention to engage and engagement behavior helps to improve user experience and interaction effectiveness [9,113]. This also has a positive effect on the design of well-being chatbots in terms of enhancing user engagement [114,115]. Therefore, the intention to engage influences engagement and is an important factor in the study of user engagement with well-being chatbots. We assume that intention to engage with AI-based well-being chatbots will be positively related to engagement behavior (hypothesis 11).

Age, gender, education and chronic disease status were implemented in the research model as control variables [116].

## Methods

### Ethical Considerations

Approval was obtained from the NOVA Information Management School Ethics Committee, NOVA University of Lisbon (INFSYS2023-5-257970). The procedures used in this study adhere to the tenets of the Declaration of Helsinki. All participants were aged at least 18 years, and informed consent was obtained from them. All data were collected anonymously, and participants were not compensated.

### Data Collection and Sample

The questionnaire was developed in English on the Qualtrics platform. The survey was designed per the guidelines and the Checklist for Reporting Results of Internet E-Surveys (CHERRIES), which is presented in Multimedia Appendix 1 [117]. We explained to participants that participation was voluntary, and their data would be collected anonymously. We took measures to ensure that participants clearly understood what a well-being chatbot entails by introducing its concept and benefits at the start of the survey. Meanwhile, we described the functionality and use of an AI-based well-being chatbot. We engaged 2 experts and 2 colleagues to review and evaluate the questions to ensure that the topics were clear, relevant to the subject matter, and easy to understand. Once the questionnaire was finalized, a translator translated the questions into Chinese. Then, another translator was responsible for doing a back-translation and comparing it with the original English version to ensure accuracy [118]. Then, 40 participants were selected for pretesting to validate the questions’ understandability and the survey scale. No issues were reported that could indicate that the survey items were unreliable. Action was taken to prevent potential issues with single source and common source bias. The questionnaire was placed in 3 different web platforms to ensure the maximum coverage and avoid a single-source bias [117,119,120].

We distributed the survey on 3 popular social media mobile apps: WeChat, Weibo, and Douban. WeChat is China’s most popular social media network, with 1.3 billion active users in 2022 [121]. Weibo is China’s second-largest social platform after WeChat, with 582 million active users at the end of the first quarter of 2022 [122]. Douban is an interest-oriented social network community with 75 million users as of 2020 [123,124]. Publishing the questionnaire across the 3 social media platforms will ensure fair data collection.

The framework’s independent and dependent variable items were collected in a single questionnaire. We assessed if there was a clear understanding of what was being measured by the constructs, to avoid the risk of common source bias [119,120]. The aim was for the respondents to avoid using the same mental process or heuristics when replying to questions about different constructs [119,120,125]. The assessment of our pilot survey was that there was no reason for concern. For added precaution, additional features were incorporated in the final survey to enable psychological separation. While designing a survey, psychological separation should ensure that the measures of the different constructs are unrelated [120]. Different instructions for different sections of the survey were provided, and the sections of the survey that measure different constructs were physically separated [120].

Finally, 256 valid replies from well-being chatbot users were collected from May to October 2023. The web-based survey did not impose any restrictions on participants other being an adult aged ≥18 years.

### Measurement

The scales of all the variables in this study were produced concerning the relevant literature. Minor modifications were carried out according to the characteristics of AI-based chatbots. We used a 7-point scale to assess the variables from 1=“strongly disagree” to 7=“strongly agree.” The questionnaire with the measurement items and references for each variable are provided in Multimedia Appendix 2.

### Data Analysis

The data were analyzed using the partial least squares structural equation modeling (PLS-SEM) approach using Smart-PLS (version 4.0) [126], which is suitable for analyzing and predicting complex models and nonnormally distributed data. PLS-SEM can also handle models that include both reflective and formative variables [127].

Reflective and formative construct measurements were included in the research. In reflective measurement models, causality flows from the underlying construct to the indicator. In contrast, in formative measurement models, causality flows in the opposite direction, from indicators to constructs [128]. Reflective constructs measure entities with a series of positively correlated items [129,130]. In contrast, the formative construct is a singular construct which is constituted by the aggregation of multiple indicators without any a priori assumptions regarding the interrelationships between these elements [129,130]. Reflective and formative measurement models should be evaluated separately [128].

## Results

### Sample Characteristics

Of the 256 valid samples, all had experience in using AI-based chatbot for mental health care. The participants’ average age was 30.9 years, and 55.9% of participants were younger than 30 years. The average age in other studies in China with the same scope as this study has ranged between 21 and 34.8 years [25-28]. The high proportion of young women was also present in demographic data from other studies, particularly studies on health technology adoption behaviors [109,131,132]. A recent Chinese study from 2023 showed that 77% of users of digital mental health technologies in China were female [28], which aligns with our study participants’ demographics. Approximately 91% of the participants held higher education degrees, which is more prevalent in innovation technology adoption studies [133,134]. However, the number of participants with chronic diseases was close to that of those without any disease, which is also reflected in the results of previous studies, which have found that chronic diseases have an impact on health applications [116]. The sample characteristics are shown in Table 1.

**Table 1:** Demographic data (n=256).

Characteristics		Participants, n (%)
**Age (years)**		
	18-29	143 (56)
	30-44	84 (33)
	45-59	13 (5)
	≥60	16 (6)
**Gender**		
	Women	187 (73)
	Men	69 (27)
**University education**		
	Degree	234 (91)
	No degree	22 (9)
**Chronic disease status**		
	Yes	111 (43)
	No	145 (57)

### Measurement Model

Formative and reflective constructs were included in our model. They were measured separately. First, for reflective constructs, the construct items’ reliability was assessed by computing the value of each item. The loading values of all reflective construct items were above the threshold of 0.7, and they were accepted [135] and are listed in Multimedia Appendix 3. Then, we applied the Cronbach α reliability coefficient and composite reliability (CR) to measure their internal consistency. All Cronbach α and CR scores were above 0.7, and the model was proven to have good reliability [135]. Meanwhile, we examined convergence validity by assessing the average variance extracted (AVE); the value of AVE for each construct was >0.5 [135]. All detailed indicators for mean, SD, Cronbach α, CR, and AVE are shown in Table 2. We used the heterotrait-monotrait (HTMT) ratio as the main criterion to assess discriminant validity, following the latest guidelines recommendation [135-137]. The HTMT values were below the threshold value of 0.90 [135], thus confirming discriminant validity. The results are shown in Table 2. In addition, cross-loadings and the Fornell-Larcker criterion were also evaluated for discriminant validity, and the results also confirm discriminant validity (Multimedia Appendices 3 and 4).

**Table 2:** Indicators of reflective constructs.

Construct	Values, mean (SD)	Cronbach α	CR^a^	AVE^b^	HTMT^c^ values										
					PC^d^	Affect	Habit	FC^e^	Trust	Compatibility	Complexity	RA^f^	RD^g^	ITE^h^	EB^i^
PC	4.726 (1.306)	.884	.885	.742	—^j^	—	—	—	—	—	—	—	—	—	—
Affect	4.638 (1.533)	.939	.942	.890	.641	—	—	—	—	—	—	—	—	—	—
Habit	4.333 (1.329)	.822	.828	.651	.720	.695	—	—	—	—	—	—	—	—	—
FC	4.770 (1.269)	.846	.846	.684	.684	.602	.573	—	—	—	—	—	—	—	—
Trust	4.461 (1.280)	.893	.896	.703	.839	.702	.768	.614	—	—	—	—	—	—	—
Compatibility	4.508 (1.344)	.851	.853	.771	.725	.746	.732	.666	.767	—	—	—	—	—	—
Complexity	4.921 (1.292)	.896	.900	.763	.690	.679	.648	.811	.697	.791	—	—	—	—	—
RA	4.663 (1.242)	.849	.855	.689	.816	.707	.768	.676	.842	.805	.752	—	—	—	—
RD	4.620 (1.362)	.856	.858	.777	.691	.659	.659	.685	.753	.843	.837	.836	—	—	—
ITE	4.575 (1.415)	.894	.894	.825	.728	.726	.776	.771	.759	.766	.701	.766	.720	—	—
EB	4.567 (1.362)	.896	.896	.763	.779	.723	.803	.653	.827	.758	.662	.760	.736	.897	—

^a^CR: composite reliability.

^b^AVE: average variance extracted.

^c^HTMT: heterotrait-monotrait.

^d^PC: perceived consequences.

^e^FC: facilitating conditions.

^f^RA: relative advantages.

^g^RD: results demonstrability.

^h^ITE: intention to engage.

^i^EB: engagement behavior.

^j^Not applicable.

Social factors (SF) were measured as a formative construct in our research model [53]. We assessed the collinearity among indicators of the formative construct by calculating the variance inflation factor (VIF). The VIF values (Table 3) were below the cutoff value of 5 [135], which meant that there was no collinearity. Finally, we implemented a bootstrapping approach with 5000 resamples for identifying the statistical significance of each path. Social factor (SF) 1 and SF2 present statistically significant outer weights; SF3, SF4 and SF5 did not present statistically significant outer weights. Thus, we verified the SF3, SF4 and SF5 outer loading values, which were all >0.5 [135]. This aspect means that all SF items were relevant.

**Table 3:** Indicators of formative construct.

SF^a^	VIF^b^	Outer weights	*P* values (outer weights)	Outer loadings	*P* values (outer loadings)
SF1	2.386	.224	.04	.826	<.001
SF2	2.411	.421	<.001	.881	<.001
SF3	2.441	.253	.07	.834	<.001
SF4	2.841	.111	.37	.781	<.001
SF5	2.520	.198	.13	.738	<.001

^a^SF: social factor.

^b^VIF: variance inflation factor.

In addition, we used Harman 1-factor test method to probe for common method variance (CMV).

The total variance extracted by 1 factor was less than the recommended threshold of 50%. Hence, this data should not present any problem with CMV [120]. Afterward, the marker variable technique was adopted to assess the CMV, and an unrelated construct was defined as a marker variable to determine the relationship between it and each construct in the research model [125]. We obtained 0.055 (5.5%) as the maximum shared variance with other variables. Therefore, the value can be considered low [138]. After verification by 2 methods, it was concluded that the influence of CMV can be excluded from this study [120,125].

### Structural Model

**Figure 2:**
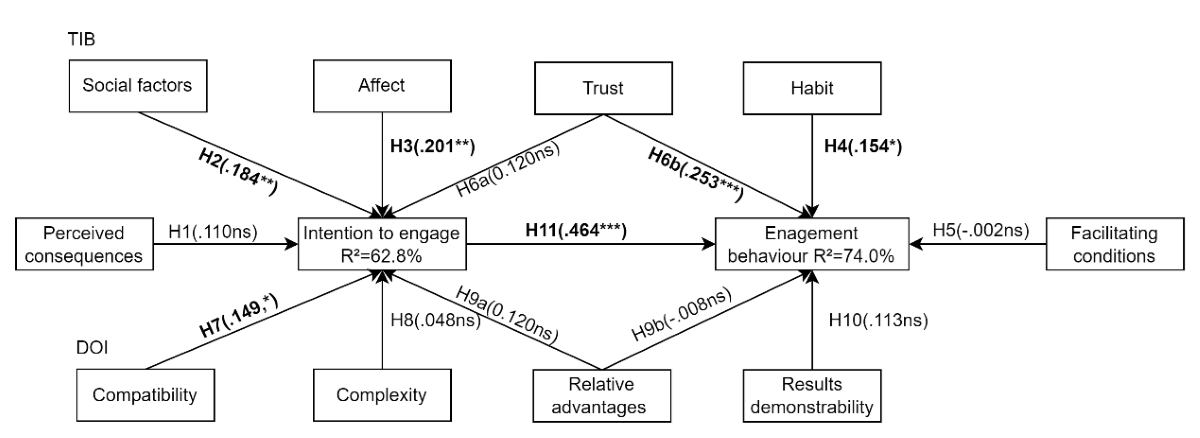
Structural model results. DOI: diffusion of innovation; H: hypothesis; ns: nonsignificant; TIB: theory of interpersonal behavior. *P<.05; ** *P*<.01; ***P<.001.

The structural model explained 62.8% variance in intention to engage, and 74% of variance in engagement behavior. Both *R*^2^ are regarded as high by the literature [135]. High *R*^2^ values indicate that our key target variables can be well predicted via the PLS path model [135]. Figure 2 shows the structural model results and identifies which latent variables are statistically significant.

Regarding the intention to engage, affect (β=.201; *P=*.002), SF (β=.184; *P=*.007), and compatibility (β=.149; *P=*.03) were statistically significant. Hypotheses 2, 3, and 7 were supported. Perceived consequences (β=.110; *P=*.12), trust (β=.120; *P=*.11), complexity (β=.048; *P=*.47), and relative advantages (β=.120; *P=*.16) were not statistically significant; hence hypotheses 1, 6a, 8, and 9a were not supported.

About engagement behavior, intention to engage (β=.464, *P<*.001), habit (β=.154; *P=*.01) and trust (β=.253, *P<*.001) were statistically significant, and facilitating conditions (β=−.002; *P=*.98), relative advantages (β=−.008; *P=*.91), and results demonstrability (β=.113; *P=*.10) were not statistically significant. Hypotheses 11, 6b, and 4 were supported, and hypotheses 5, 9b, and 10 were rejected, as shown in Table 4.

**Table 4:** Hypothesized path analysis.

Hypothesis	Path	β	*P* values	Supported
H1	Perceived consequences → intention to engage	.110	.12	No
H2	Social factors → intention to engage	.184	.007	Yes
H3	Affect → intention to engage	.201	.002	Yes
H4	Habit → engagement behavior	.154	.01	Yes
H5	Facilitating conditions → engagement behavior	−.002	.98	No
H6a	Trust → intention to engage	.120	.11	No
H6b	Trust → engagement behavior	.253	<.001	Yes
H7	Compatibility → intention to engage	.149	.03	Yes
H8	Complexity → intention to engage	.048	.47	No
H9a	Relative advantages → intention to engage	.120	.16	No
H9b	Relative advantages → engagement behavior	−.008	.91	No
H10	Results demonstrability → engagement behavior	.113	.10	No
H11	Intention to engage → engagement behavior	.464	<.001	Yes

The PLSpredict algorithm was used to assess the framework predictive power. The method uses training and hold out samples to generate and evaluate predictions from PLS path model estimations [139,140]. The guideline recommendation was followed, and the number of folds was set to 10 [140]. This approach was done because it is possible to achieve a statistical power of 80% to detect minimum *R*^2^ values of 0.1 in the endogenous constructs in the structural model for a significance level of 1% [137,140]. The first parameter to be evaluated was the Q^2^_predict_ of the indicators concerning our endogenous variables that was above 0, showing that the model demonstrates predictive power [140]. To evaluate the predictive magnitude, we compared the PLS-SEM study model with the naive linear regression model (LM) to see if it can outperform the LM benchmark [140]. Because the prediction errors distribution was considerably asymmetrical, with high kurtosis values (>1) [135], the mean absolute error was the more appropriate prediction statistic [140].

The PLS-SEM analysis yielded lower prediction errors for most of the dependent variables’ indicators, as seen in Table 5; this indicates a medium predictive power for the study model [140]. When complex models are used, involving several theories, such as those explaining human behavior, *R*^2^ values higher than 0.5 can be regarded as substantial [135,137]. The model in this research study is complex, and achieving medium predictive power is challenging in such models [137,140]. Given that this research model shows a substantial *R*^2^ for a complex model and medium predictive power, it provides confidence in its use for real-world applications [135,137,140].

**Table 5:** Prediction summary.

Indicators	Q^2^_predict_	PLS_SEM_RMSE^a^	PLS_SEM_MAE^b^	LM_RMSE^c^	LM_MAE^d^	Kurtosis	Skewness
EB1^e^	.431	1.165	.844	1.149	.810	2.029	−.542
EB2	.461	1.157	*.863* ^f^	1.196	.886	.673	−.048
EB3	.490	1.114	*.811*	1.230	.913	1.550	−.152
EB4	.557	1.057	*.776*	1.157	.831	2.030	−.413
ITE1^g^	.485	1.128	.835	1.071	.785	2.374	−.461
ITE2	.472	1.135	*.814*	1.167	.869	2.782	−.740
ITE3	.452	1.156	*.861*	1.189	.884	1.862	−.627

^a^PLS_SEM_RMSE: partial least squares structural equation modeling root mean squared error.

^b^PLS_SEM_MAE: partial least squares structural equation modeling mean absolute error.

^c^LM_RMSE: linear regression model root mean squared error.

^d^LM_MAE: linear regression model mean absolute error.

^e^EB: engagement behavior.

^f^Most relevant errors to define the model predictive power are highlighted in italics.

^g^ITE: intention to engage.

## Discussion

### Principal Findings

The TIB was considered in this study, and it was extended with the DOI theory and trust to explore in depth the factors influencing users’ engagement behavior with AI-based chatbots for well-being. The new extended model was well explained, with *R*^2^ values of 62.8% for the variance in intention to engage and 74% for the variance in engagement behavior. RQ1 was answered in this study. AI-based well-being chatbots are gradually beginning to play an active role in the field of mental health. In this context, this study obtained important results, including affect, habit, SF, trust, compatibility and intention to engage as determinants influencing users’ engagement behavior. By extending the TIB to include important research variables in the model, a foundation was laid for future, related theoretical research and practice. The study’s results affirmed the significance of affect on users’ intention to engage. Our measurement of user affect contains both positive and negative emotions [141]. From the user’s perspective, there is a willingness to engage with the chatbot. It indicates that users have a positive impression of the well-being chatbot’s service and that users are inclined to deal with their emotions by engaging with a well-being chatbot. Users are beginning to be comfortable with the service as a mental health intervention that is available anytime, anywhere, without an appointment [142]. From the perspective of well-being chatbot characteristics, empathetic chatbots can understand users’ emotions and provide professional psychological counseling or companionship [96]; for instance, suggesting meditation, outdoor activities, or socializing with friends [7,143,144]. This proactive intervention on users’ emotions promotes users’ willingness to participate with sustained engagement behaviors.

Habit has been extensively studied in previous research on health app use and engagement behavior [145]. In the current information age, smartphone use has become ingrained in people’s daily lives. Engaging with mobile apps for specific purposes has become a habit for many individuals [146]. This study confirmed that habits were an essential factor influencing users’ engagement behavior, aligning with other studies’ findings [147]. In addition, the significant influence of SF indicated that the opinions of friends, family, and medical professionals substantially impacted individuals’ intention to engage with well-being chatbots. This finding is consistent with previous studies [78].

Furthermore, as AI-based well-being chatbots represent an innovative technology in a digital mental health intervention, proactively exploring methods for promoting their adoption is worthwhile. This research model incorporates features from the DOI framework to extend the TIB model. Among these features, compatibility was found to impact the intention to engage significantly, while the other 3 aspects did not demonstrate statistical significance. Because chatbots work like any other social software, the experience is consistent with past experiences [148,149]. They can be used easily and without extra effort, making users more willing to use them, which is also consistent with previous research [150].

Trust, habit, and intention to engage were statistically significant for chatbot engagement. They are both internal (subjective) elements of individuals [151,152], whereas other factors had no statistically significant influence, such as facilitating conditions, relative advantages, and results demonstrability. It indicated that the user’s subjective sense of experience played a decisive role in engagement compared with other factors [153,154]. This suggests that a lack of user-centeredness in product design or a lack of information about mental health services in terms of content that meets users’ needs could directly reduce user engagement [155]. Consistent with the findings of this study, it further underlines the importance of user-centeredness.

Potential explanations for the constructs that were nonsignificant are also addressed. The facilitating conditions hypothesis was not supported, aligning with previous research [32,156]. When engaging with an AI-based well-being chatbot, users’ ability to access smartphones, computers, or the internet, as well as their knowledge of how to use them, did not become barriers—most likely because our respondents were young and highly educated [157]. It could have been expected that complexity would be statistically significant; nonetheless, other studies of new technologies in health care, when complexity was evaluated as part of DOI, also obtained nonsignificant results [13,91,94,157-160]. A possible explanation, also supported by the literature, is that early adopters of new technologies, as in the case of our study, have a higher cognitive ability and are more accustomed to managing complexity, so they do not perceive complexity as an obstacle to using new technologies, including AI-based well-being chatbots [13,91,94,157,160]. Some research indicates that relevant advantages could influence users’ willingness to adopt new technologies in the initial implementation phase [161,162]. However, not all studies support this. The nonconfirmation of the relative advantages hypothesis in our research suggests that individuals focused more on the experiences and value derived from chatbots’ services rather than the direct benefits of outcomes [163]. The emotional component—affect—is much more relevant than technical factors, minimizing their impact on the model. Among younger people with higher level of education, perceived consequences show different influences on behavioral intention [164]. In our study, perceived consequences had a nonsignificant impact on the intention to engage with a well-being chatbot, suggesting that individuals become more focused on their engaging experience rather than on the positive or negative consequences.

Age, gender, education, and chronic disease status as control variables were not statistically significant for the 2 dependent variables (intention to engage and engagement behavior) in the model. Our study respondents were young, were mostly highly educated, included a high proportion of women, and had a ratio of respondents with chronic diseases aligned with the literature [25-28,116]. In addition, early adopters exhibit behavior toward a technology that differs from those who adopt it later [94,157,160]. AI-based well-being chatbots are a new technology, currently being used by early adopters with no significant heterogeneity, which, at this early stage of implementation, does not contribute to significant results for the control variables.

### Theoretical Implications

First, we provided an integrated perspective of TIB, DOI and trust to uncover the critical factors influencing chatbot intention to engage and engagement and how these factors influence individual decision-making. In previous studies, the technology acceptance model and unified theory of acceptance and use of technology were considered the most used and integrated information systems theories, and there were few relevant adoptions of TIB [165]. However, we believed that affect and habit in TIB were most appropriate for explaining AI-based well-being chatbot use behavior based on its characteristics. The new integrated model provides important theoretical support for future research on AI-based chatbots and other products.

Trust is among the most important factors determining human intention to adopt smart technology products [166]. Adding trust to the research model explained users’ intention to engage and engagement behavior in a multidimensional way. The results of this study provided empirical evidence for the completeness of future AI information system application models. Nowadays, AI technology is being increasingly applied in diverse information systems and the autonomy of information systems has been enhanced. Theoretically validating the impact of trust on AI products is imperative.

### Practical Implications

The results of our study model can provide guidance on better implementing AI-based well-being chatbots to increase their adoption. The triangulation between users, companies that develop AI-based well-being chatbots, and mental health practitioners is highly relevant and should be considered during the system’s development and real-world use. In our model, affect had a statistically significant impact on the intention to engage with AI-based well-being chatbots. It is crucial for designers and developers to recognize the influence of users’ affect on their willingness to engage with AI-based well-being chatbots. Affect describes a wide range of feelings, both positive and negative [72]. Developers should address the needs of users in different emotional states during the design and development process while simultaneously enhancing the system’s ability to recognize emotions and build emotional connections between chatbots and users [167,168]. Because affect is a complex dimension that transcends the purely IT aspects of the chatbot, developers should seek the support of mental health practitioners from the early stages of these systems’ development. Habit is also a statistically significant construct, so providing good support services should be a key area of focus for AI-based well-being chatbot companies, as they contribute to user experience and help maintain users’ habits of continuous engagement with the chatbot. According to our model results, SF also contribute to the intention to engage AI-based well-being chatbots. It suggests that individuals relevant to users can influence the adoption of AI-based well-being chatbots [51]. This indicates that mental health practitioners may influence users’ intention to engage with AI-based well-being chatbots. For companies developing AI-based well-being chatbots, it is important to engage with mental health practitioners. Developers could also leverage social media to promote their AI-based well-being chatbots, considering the relevance of SF.

Trust was another statistically significant construct in the model. Establishing user trust is pivotal for driving participation behavior, and devising strategies to cultivate user trust requires careful consideration. They should consider introducing mental health practitioners into the system development process [169]. The incorporation of mental health practitioners enhances the efficacy and competitive advantage of chatbots, while reinforcing user trust and fostering their intention to engage. In some current practices, mental health experts have already been involved in the chatbot development process, with positive outcomes [170].

Another relevant construct in the model is compatibility. It is recommended that users be included in the entire life cycle of the AI-based well-being chatbot, as this allows a comprehensive understanding of users’ habits and lifestyles, thereby facilitating product compatibility [8,171]. By communicating with users in greater depth, it is possible to gain insights into their interactions with the chatbot and to establish trust. The emotional connection between AI-based well-being chatbots and their users represents the core value of this technology, requiring designers and developers to prioritize a user-centered approach in their work [172].

Although this research did not cover older people or those with special needs, future developments specifically targeting these groups should account for the fact that they may not have access to certain resources or may have lower digital literacy [173]. In these cases, the constructs of complexity and facilitating conditions, which were nonsignificant in this research, should play a critical role. Less complex systems and providing the right resources should increase the adoption of AI-based well-being chatbots within these groups.

**Figure 3:**
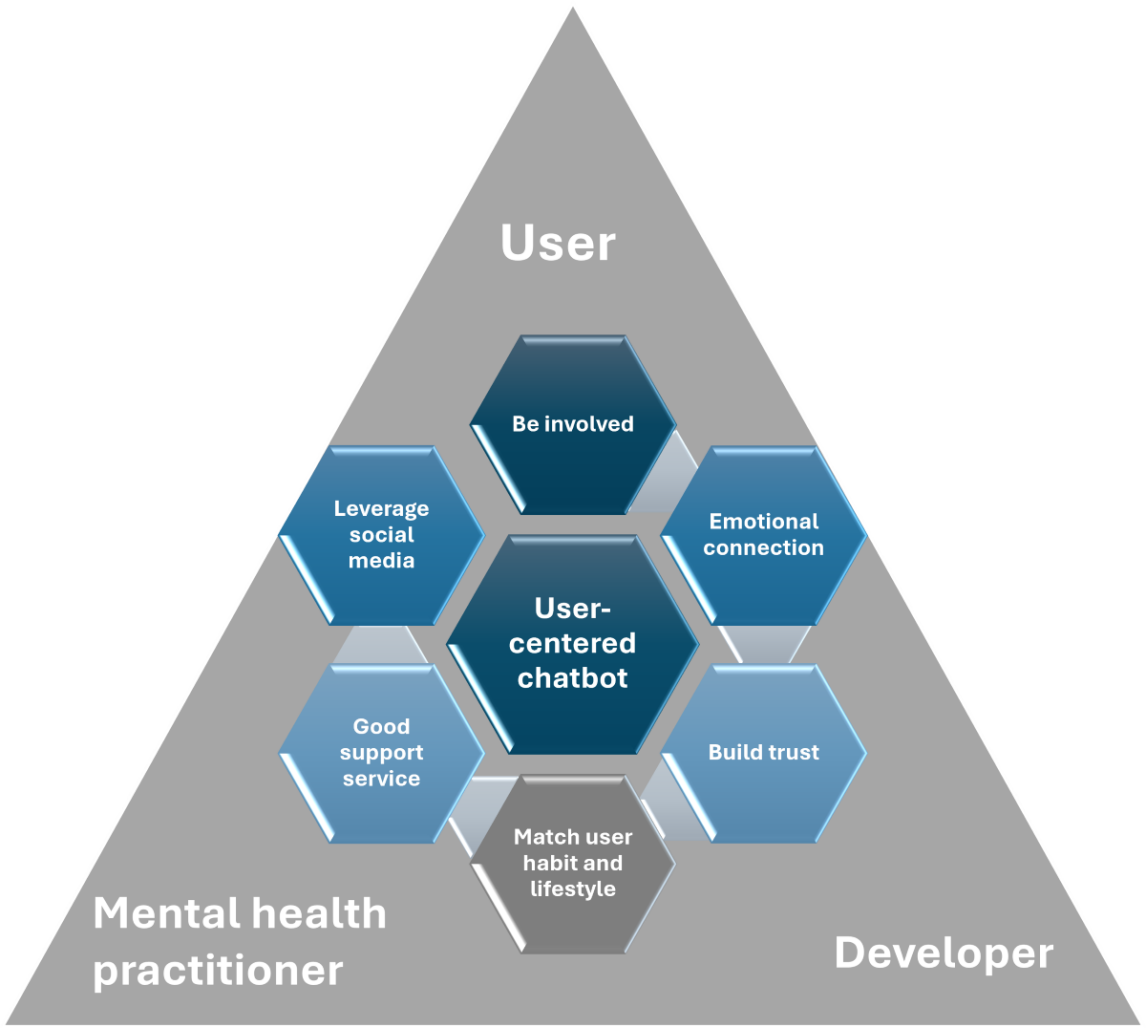
Suggested practical implications.

The involvement of mental health practitioners and users, integrating and applying their feedback throughout the AI-based well-being chatbot’s development, is essential for successfully implementing the chatbot’s entire life cycle. It is crucial to use AI-based well-being chatbots for therapeutic purposes under the guidance of qualified professionals to prevent misuse, which could potentially result in risky behaviors [174,175]. Figure 3 provides a graphical representation of the suggested practical implications. RQ2 about improving user experience and engagement from a practical point of view, leading to enhanced service levels was answered.

### Limitations and Future Research

Our study used a convenience sample for which an online, multiplatform collection approach with a large coverage of the Chinese population was used to prevent single-source bias [120]. Still, the approach was not entirely random because we only posted the questionnaire, making it available, and we did not send messages to all platform users. In addition, the data source was from only one country. Future studies could use probabilistic sampling approaches. Access to large databases of AI-based well-being chatbot users could support studies with random sampling. Future research should expand the data sources to include participants from different countries and ethnicities, as well as special target populations (eg, people diagnosed with depression, older people). A multicountry approach as the next step may be used to evaluate if the findings are generalizable. Future studies could also collect the independent and dependent variables in different moments in time to reduce the probability of common-source bias [120]. Collection of real use data from the AI-based well-being chatbots could also be an advantage in future studies. Specific criteria for measuring engagement, such as the number of minutes of participation in using the chatbot, the number of logins, and the number of completed modules were not counted [176]. A future study may also explore engagement with AI-based well-being chatbots from mental health practitioners’ perspective, as well as conduct qualitative research or quantitative research.

### Conclusions

AI-based well-being chatbots provide users with emotional support to help alleviate conditions such as loneliness and anxiety. They are an effective solution to the lack of resources for mental health care. Exploring the factors affecting their use carries great significance. This paper extended past models by using DOI and trust theory, based on TIB. It proposed an integrated model that effectively explained the factors affecting individuals’ acceptance and engagement with AI-based well-being chatbots. Among them, affect, habit, and trust play vital roles. The important theoretical role of the TIB model in the context of chatbots was validated. In addition, recommendations for the design of well-being chatbots were presented. For example, human-centered design concepts, attention to ethical issues, and building trust through characterization have important practical implications.

## Appendix

Multimedia Appendix 1 Checklist for Reporting Results of Internet E-Surveys (CHERRIES) checklist.

Multimedia Appendix 2 Study questionnaire.

Multimedia Appendix 3 Outer loadings and cross-loadings.

Multimedia Appendix 4 Fornell-Larcker criterion.

## Abbreviations

ABC: attitude-behavior-context
AI: artificial intelligence
AVE: average variance extracted
CR: composite reliability
DOI: diffusion of innovation
HTMT: heterotrait-monotrait
ITE: intention to engage
mHealth: mobile health
PLS-SEM: partial least squares structural equation modeling
SF: social factors
TIB: theory of interpersonal behavior
VIF: variance inflation factor
