# Supplementary material for: A New Research Model for Artificial Intelligence–Based Well-Being Chatbot Engagement: Survey Study
Source: JMIR Hum Factors. 2024 Nov 11;11:e59908. doi: 10.2196/59908 (PMC11589509; doi:10.2196/59908)
Supplement: Multimedia Appendix 1 [file humanfactors_v11i1e59908_app1.pdf]

Multimedia Appendix 1. Checklist for Reporting Results of Internet E-Surveys (CHERRIES)

Checklist Item	*Explanation and answers*
Describe survey design	Describe target population, sample frame. Is the sample a convenience sample? (In “open” surveys this is most likely.)<br><br>Target population:<br>To investigate the factors that influence user’s engagement with well-being chatbots, our target population should simultaneously meet two criteria.<br>1. Individuals over the age of 18 who are the users of internet or smartphone.<br>2. Individuals who currently use well-being chatbots or those who had use experience before.
IRB approval	Mention whether the study has been approved by an IRB.<br><br>Approval was obtained from the NOVA Information Management school ethics committee, NOVA University of Lisbon. The procedures used in this study adhere to the tenets of the Helsinki Declaration. All subjects were at least 18 years old and informed consent was obtained from them. The project relied on the use of anonymous information.
Informed consent	Describe the informed consent process. Where were the participants told the length of time of the survey, which data were stored and where and for how long, who the investigator was, and the purpose of the study?<br><br>Upon opening the survey link, the initial section includes an overview of the study's objectives and a brief description of its contents, as well as a statement indicating that the study is conducted by NOVA IMS researchers and that all data is used for academic purposes and in accordance with NOVA IMS data regulations.<br>Furthermore, the project is voluntary and anonymous. It will take approximately 15-20 minutes. If participants have read the above information, voluntarily agree to participate and be at least 18 years old, agree button was chosen and move to the questionnaire interface; If participant choose disagree button, they will directed to the survey’s end p age.

Data protection	If any personal information was collected or stored, describe what mechanisms were used to protect unauthorizedaccess.<br><br>The survey was anonymous, participants' names, address, email and contact information were not collected.
Development and testing	State how the survey was developed, including whether the usability and technical functionality of the electronic questionnaire had been tested before fielding the questionnaire.<br><br>All questionnaires' Items were developed with reference to questionnaire content from previous studies; minor adjustments were made to fit the context of this study. The Qualtrics platform was used to create the questionnaire. Forty questionaries were collected for pre-testing. The results confirmed that there were no problems with the readability and content of the questionnaire and ensured that there were no technical problems on Qualtrics. The online questionnaire was posted on major social media.

Open survey versus closed survey	An “open survey” is a survey open for each visitor of a site, while a closed survey is only open to a sample whichthe investigator knows (password-protected survey).<br><br>The questionnaire was distributed in an open-access mode. Anyone can open the online questionnaire.
Contact mode	Indicate whether or not the initial contact with the potential participants was made on the Internet. (Investigatorsmay also send out questionnaires by mail and allow for Web-based data entry.)<br><br>The investigator did not contact potential participants in any way.
Advertising the survey	How/where was the survey announced or advertised? Some examples are offline media (newspapers), or online (mailing lists – If yes, which ones?) or banner ads (Where were these banner ads posted and what did they look like?). It is important to know the wording of the announcement as it will heavily influence who chooses to participate. Ideally the survey announcement should be published as an appendix.<br><br>The investigator posted the survey in the relevant group which contained the topic of well-being chatbot and personal page on social media.
Web/E-mail	State the type of e-survey (eg, one posted on a Web site, or one sent out through e-mail). If it is an e-mail survey,were the responses entered manually into a database, or was there an automatic method for capturing responses?<br><br>The online survey was posted on Weibo, Wechat and Douban which were three popular social media platforms. Responses were captured automatically.

Context	Describe the Web site (for mailing list/newsgroup) in which the survey was posted. What is the Web site about, who is visiting it, what are visitors normally looking for? Discuss to what degree the content of the Web site couldpre-select the sample or influence the results. For example, a survey about vaccination on a anti-immunization Web site will have different results from a Web survey conducted on a government Web site<br><br>We used three social media platform or application to publish our survey.<br>1.WeChat is China's most popular social media network, with 1.3 billion active users until 2022. WeChat enables real-time communication between users on a one-to-one basis as well as within groups on different topics. Users can post updates such as pictures, videos or links in their personal moments and friends can interact with them based on the updates.<br><br>2.Weibo is China's second-largest social platform after WeChat, with 582 million active users at the end of the first quarter of 2022. Weibo enables real-time communication between users. User can publish updates and all users of Weibo can access it and interact with the user.<br><br>3.Douban is an interest-oriented social network community with 75 million users as of 2020.Douban works same as Weibo.
Mandatory/voluntary	Was it a mandatory survey to be filled in by every visitor who wanted to enter the Web site, or was it a voluntarysurvey?<br><br>The survey was voluntary, all participants had an option to agree or disagree to join the survey.
Incentives	Were any incentives offered (eg, monetary, prizes, or non-monetary incentives such as an offer to provide the survey results)?<br><br>No monetary incentives were offered, and respondents consented to provide their responses voluntarily.

Time/Date	In what timeframe were the data collected?<br><br>Data collection took place during May and October 2023.
Randomization of items or questionnaires	To prevent biases items can be randomized or alternated.<br><br>Yes, they were alternated.
Adaptive questioning	Use adaptive questioning (certain items, or only conditionally displayed based on responses to other items) to reduce number and complexity of the questions.<br><br>The only adaptive question was related to exclude the non-users from replying the questionnaire. Users had to reply the full questionnaire.
Number of Items	What was the number of questionnaire items per page? The number of items is an important factor for the completion rate.<br><br>There were 2 to 6 questionnaire items per page.
Number of screens (pages)	Over how many pages was the questionnaire distributed? The number of items is an important factor for the completion rate.<br><br>16 pages.

Completeness check	It is technically possible to do consistency or completeness checks before the questionnaire is submitted. Was thisdone, and if “yes”, how (usually JAVAScript)? An alternative is to check for completeness after the questionnairehas been submitted (and highlight mandatory items). If this has been done, it should be reported. All items shouldprovide a non-response option such as “not applicable” or “rather not say”, and selection of one response optionshould be enforced.<br><br>Once a participant agrees to take the survey, all survey items are mandatory to complete. With Qualtrics settings, if there are uncompleted items, the system will prompt and will not be able to proceed to the next screen.
Review step	State whether respondents were able to review and change their answers (eg, through a Back button or a Reviewstep which displays a summary of the responses and asks the respondents if they are correct).<br><br>During completion, respondents were able to navigate the survey througha ‘Back’ button, which let them review and change their answers before final submission.
Unique site visitor	If you provide view rates or participation rates, you need to define how you determined a unique visitor. Thereare different techniques available, based on IP addresses or cookies or both.<br><br>We didn’t define view rated or participation rates.
View rate (Ratio of unique survey visitors/unique site visitors)	Requires counting unique visitors to the first page of the survey, divided by the number of unique site visitors(not page views!). It is not unusual to have view rates of less than 0.1 % if the survey is voluntary.<br><br>View rate was not reported.

Participation rate (Ratio of unique visitors who agreed to participate/unique first survey page visitors)	Count the unique number of people who filled in the first survey page (or agreed to participate, for example by checking a checkbox), divided by visitors who visit the first page of the survey (or the informed consents page, ifpresent). This can also be called "recruitment" rate.<br><br>Recruitment rate was not reported.
Completion rate (Ratio of users who finished the survey/users who agreed to participate)	The number of people submitting the last questionnaire page, divided by the number of people who agreed to participate (or submitted the first survey page). This is only relevant if there is a separate "informed consent" page or if the survey goes over several pages. This is a measure for attrition. Note that "completion" can involveleaving questionnaire items blank. This is not a measure for how completely questionnaires were filled in. (If youneed a measure for this, use the word "completeness rate".)<br><br>Completion rate was not reported.
Cookies used	Indicate whether cookies were used to assign a unique user identifier to each client computer. If so, mention thepage on which the cookie was set and read, and how long the cookie was valid. Were duplicate entries avoided by preventing users access to the survey twice; or were duplicate database entries having the same user ID eliminated before analysis? In the latter case, which entries were kept for analysis (eg, the first entry or the mostrecent)?<br><br>Cookies were not used.

IP check	Indicate whether the IP address of the client computer was used to identify potential duplicate entries from the same user. If so, mention the period of time for which no two entries from the same IP address were allowed (eg,24 hours). Were duplicate entries avoided by preventing users with the same IP address access to the survey twice; or were duplicate database entries having the same IP address within a given period of time eliminated before analysis? If the latter, which entries were kept for analysis (eg, the first entry or the most recent)?<br><br>IP check was used by Qualtrics, we use a system function to prevent multiple submissions through Qualtrics.
Log file analysis	Indicate whether other techniques to analyze the log file for identification of multiple entries were used. If so,please describe.<br><br>We use a system function to prevent multiple submissions through Qualtrics.
Registration	In "closed" (non-open) surveys, users need to login first and it is easier to prevent duplicate entries from the sameuser. Describe how this was done. For example, was the survey never displayed a second time once the user had filled it in, or was the username stored together with the survey results and later eliminated? If the latter, whichentries were kept for analysis (eg, the first entry or the most recent)?<br><br>Our survey is an open survey. IP check was used by Qualtrics, we use a system function to prevent multiple submissions through Qualtrics.
Handling of incomplete questionnaires	Were only completed questionnaires analyzed? Were questionnaires which terminated early (where, forexample, users did not go through all questionnaire pages) also analyzed?<br><br>Our analysis involved completed questionnaires only. We didn't analyze incomplete parts.

Questionnaires submitted with an atypical timestamp	Some investigators may measure the time people needed to fill in a questionnaire and exclude questionnaires thatwere submitted too soon. Specify the timeframe that was used as a cut-off point, and describe how this point wasdetermined.<br><br>Timestamp was not used.
Statistical correction	Indicate whether any methods such as weighting of items or propensity scores have been used to adjust for thenon-representative sample; if so, please describe the methods.<br><br>No such methods were used.
