# Supplementary material for: A New Research Model for Artificial Intelligence–Based Well-Being Chatbot Engagement: Survey Study
Source: JMIR Hum Factors. 2024 Nov 11;11:e59908. doi: 10.2196/59908 (PMC11589509; doi:10.2196/59908)
Supplement: Multimedia Appendix 2 [file humanfactors_v11i1e59908_app2.pdf]

Appendix 2. Study questionnaire (English version).

Content and constructs	Questions and reference
Introduction	Dear Participants,<br><br>Thank you for participating in this survey.<br><br>This is a study related to the use of a well-being chatbot application. Well-being chatbots provide customers with emotional support chat services in the form of mobile apps or web platforms, relying on artificial intelligence technology. It provides online feedback in real time when you need it, offering professional advice in relevant areas, especially dealing with health advice such as mood issues (e.g., Woebot, ChatGPT). This survey is conducted for people who have experience in interacting with chatbots for well-being or mental health.<br><br>Your participation in this research study is voluntary. Your responses will be confidential and anonymous. The results of this study will be used for academic purposes only. The survey consists entirely of multiple-choice questions and takes 15-20 minutes to complete. We are researchers from NOVA IMS, if you have any questions, you can contact us (20220021@novaims.unl.pt). Thank you again for your participation.<br><br>ELECTRONIC CONSENT: Please select your choice below.<br><br>Clicking on the "agree" button below indicates that:<br>• you have read the above information<br>• you voluntarily agree to participate<br>• you are at least 18 years old<br><br>Please choose Agree or Disagree. (If selection is Disagree, the questionnaire ends)
Q1	Are you a well-being chatbot user? (If selection is No, the questionnaire ends)<br>• Yes<br>• No
Q2	What's your gender?<br>• Female<br>• Male<br>• Other
Q3	What is your age?<br>• 18-29<br>• 30-44<br>• 45-59<br>• 60 and above 60
Q4	Do you have a university degree?<br>• Yes<br>• No
Q5	Do you have a chronic health condition?<br>• Yes<br>• No
Perceived consequences	The purpose of engaging with well-being chatbots is: (from 1 - Strongly Disagree to 7 - Strongly Agree) [1]<br>The amount of personal time I can enjoy more<br>The increase in quality of life by using well-being chatbots<br>The extent to which my life become more interesting<br>The increase in my work productivity
Social factors	In the list below as pertaining to his/her/their influence of you engaging with well-being chatbots purposes: (from 1 - Strongly Disagree to 7 - Strongly Agree) [2]<br>My family's influence<br>My friends' influence<br>My doctors' influence<br>My psychologist's influence<br>My nurse's influence

Affect	By engaging with a well-being chatbot: (from level 1 - Strongly Disagree to 7 - Strongly Agree) [3]<br>I feel good when I want to talk, and a chatbot can talk with me.<br>I feel sad when I want to talk, but nobody can talk with me.<br>It makes me happy to get support from a chatbot when I need help.
Habit	Please evaluate each of the items concerning the habit to engage with a well-being chatbot: (from 1 - Strongly Disagree to 7 - Strongly Agree) [4]<br>I am used to using a well-being chatbot.<br>I do not think twice before using a well-being chatbot.<br>Sometimes, I use a well-being chatbot without thinking about whether I need it or not.<br>Using a well-being chatbot is automatic for me.
Facilitating conditions	Please evaluate each of the items in the list below regarding support for engaging with a well-being chatbot: (from 1 - Strongly Disagree to 7 - Strongly Agree) [5]<br>I have the resources necessary to engage with a well-being chatbot (such as a computer or mobile phone).<br>I have the knowledge necessary to engage with a well-being chatbot.<br>I can get help from a service provider when I have difficulties engaging with a well-being chatbot.<br>A well-being chatbot is compatible with the devices I use.
Trust	About trust, please evaluate each of the items in the list below: (from 1 - Strongly Disagree to 7 - Strongly Agree) [6]<br>I trust well-being chatbots to be reliable.<br>I trust well-being chatbots to be secure.<br>I believe well-being chatbots are trustworthy.<br>I trust well-being chatbots.<br>Even if the well-being chatbot is not monitored, I would trust them to do the job correctly.
Compatibility	Please evaluate each of the items in the list below for a well-being chatbot: (from 1 - Strongly Disagree to 7 - Strongly Agree) [7]<br>Using a well-being chatbot for online chatting is compatible with all aspects of lifestyle.<br>I think using a well-being chatbot for online chatting fits well with the way I like to chat online.<br>Using with a well-being chatbot for online chatting fits into my lifestyle.
Complexity	If engaging with well-being chatbots: (from 1 - Strongly Disagree to 7 - Strongly Agree) [8]<br>Learning how to use a chatbot would be easy for me.<br>My interaction with a chatbot would be clear and understandable.<br>I find that it would be easy to engage with a chatbot.<br>It would be easy for me to become skilful at using a chatbot.
Relative advantages	Concerning engaging with well-being chatbots: (from 1 - Strongly Disagree to 7 - Strongly Agree) [8]<br>I consider that it would be useful in my daily life.<br>It increases my chances of achieving things that are important to me.<br>It helps me accomplish healthcare-related objectives more quickly.<br>It increases my productivity.
Results demonstrability	About the results of using a well-being chatbot: (from 1 - Strongly Disagree to 7 - Strongly Agree) [8]<br>I would have no difficulty telling others.<br>I believe I can communicate to others.<br>They are apparent to me.
Intention to engage	Please evaluate each of the items in the list below for a well-being chatbot: (from 1 - Strongly Disagree to 7 - Strongly Agree) [9]<br>I intend to continue engaging with a well-being chatbot in the future.<br>I will always try to engage with a well-being chatbot in my daily life.

	I plan to continue to engage with a well-being chatbot frequently.
Engagement behaviour	Please evaluate about engaging with a well-being chatbot: (from 1 - Strongly Disagree to 7 - Strongly Agree) [10]
	I feel comfortable engaging with a well-being chatbot.
	I feel that the engagement I have with a chatbot is very human-like
	The engagement I have with a well-being chatbot is very meaningful.
	I am having fun when I am engaging with a well-being chatbot.
Additional question (Marker Variable)	Please tell us: (from 1 - Strongly Disagree to 7 - Strongly Agree)
	What is your knowledge about NOVA University Lisbon?

Reference

1. Pee LG, Woon IMY, Kankanhalli A. Explaining non-work-related computing in the workplace: A comparison of alternative models. Information & Management North-Holland; 2008 Mar 1;45(2):120–130. doi: 10.1016/J.IM.2008.01.004
2. Moody GD, Siponen M. Using the theory of interpersonal behavior to explain non-work-related personal use of the Internet at work. Information & Management North-Holland; 2013 Sep 1;50(6):322–335. doi: 10.1016/J.IM.2013.04.005
3. De Miranda Coelho JAP, Gouveia VV, De Souza GHS, Milfont TL, Barros BNR. Emotions toward water consumption: Conservation and wastage. Revista Latinoamericana de Psicologia 2016;48(2):117–126. doi: 10.1016/j.rlp.2015.09.006
4. Robinson J. Triandis' Theory of Interpersonal Behaviour in understanding software piracy behaviour in the South African context.
5. Mustafa S, Zhang W, Anwar S, Jamil K, Rana S. An integrated model of UTAUT2 to understand consumers' 5G technology acceptance using SEM-ANN approach. Scientific Reports 2022 12:1 Nature Publishing Group; 2022 Nov 21;12(1):1–19. PMID:36414788
6. Silva SC, De Cicco R, Vlačić B, Elmashhara MG. Using chatbots in e-retailing – how to mitigate perceived risk and enhance the flow experience. International Journal of Retail and Distribution Management Emerald Publishing; 2023 Feb 24;51(3):285–305. doi: 10.1108/IJRDM-05-2022-0163
7. De Cicco R, Iacobucci S, Aquino A, Alparone FR, Palumbo R. Understanding Users' Acceptance of Chatbots: An Extended TAM Approach. Lecture Notes in Computer Science (including subseries Lecture Notes in Artificial Intelligence and Lecture Notes in Bioinformatics) Springer Science and Business Media Deutschland GmbH; 2022 Apr 1;13171 LNCS:3–22. doi: 10.1007/978-3-030-94890-0_1/TABLES/4
8. Viana Pereira F, Tavares J, Oliveira T. Adoption of video consultations during the COVID-19 pandemic. Internet Interventions Elsevier; 2023 Mar 1;31:100602. doi: 10.1016/J.INVENT.2023.100602
9. Salgado T, Tavares J, Oliveira T. Drivers of Mobile Health Acceptance and Use From the Patient Perspective: Survey Study and Quantitative Model Development. JMIR mHealth and uHealth JMIR Mhealth Uhealth; 2020 Jul 1;8(7). PMID:32673249
10. Schuetzler RM, Grimes GM, Scott Giboney J. The impact of chatbot conversational skill on engagement and perceived humanness. Routledge; 2020 Jul 2;37(3):875–900. doi: 10.1080/07421222.2020.1790204
