# Supplementary material for: A New Research Model for Artificial Intelligence–Based Well-Being Chatbot Engagement: Survey Study
Source: JMIR Hum Factors. 2024 Nov 11;11:e59908. doi: 10.2196/59908 (PMC11589509; doi:10.2196/59908)
Supplement: Multimedia Appendix 3 [file humanfactors_v11i1e59908_app3.pdf]

Multimedia Appendix 3 The value of outer loading (printed with bold) and cross-loading.

	PC	SF	Affect	Habit	FC	Trust	Compatibility	CO	RA	RD	ITE	EB
PC1	**.855**	.580	.486	.508	.520	.626	.533	.503	.583	.533	.548	.589
PC2	**.892**	.543	.515	.513	.527	.646	.548	.548	.603	.528	.580	.610
PC3	**.865**	.563	.520	.557	.551	.650	.600	.581	.635	.537	.533	.594
PC4	**.833**	.600	.491	.570	.449	.647	.482	.485	.601	.474	.567	.598
SF1	.554	**.824**	.552	.552	.551	.559	.534	.609	.553	.570	.556	.546
SF2	.518	**.878**	.554	.545	.554	.559	.539	.592	.537	.594	.592	.615
SF3	.583	**.831**	.515	.563	.481	.647	.537	.541	.591	.553	.561	.562
SF4	.548	**.778**	.479	.508	.485	.599	.515	.524	.492	.484	.525	.586
SF5	.516	**.736**	.464	.480	.440	.559	.424	.443	.454	.403	.496	.547
Affect1	.566	.587	**.942**	.576	.505	.605	.633	.575	.579	.583	.672	.662
Affect2	.526	.598	**.946**	.586	.496	.585	.622	.589	.615	.551	.607	.601
Affect3	.560	.617	**.943**	.590	.519	.635	.629	.608	.600	.540	.605	.614
Habit1	.582	.560	.578	**.813**	.424	.604	.592	.474	.643	.519	.648	.638
Habit2	.486	.530	.473	**.817**	.314	.513	.456	.413	.478	.380	.513	.546
Habit3	.351	.430	.374	**.779**	.346	.422	.403	.371	.362	.391	.423	.486
Habit4	.564	.568	.547	**.819**	.479	.585	.532	.538	.585	.495	.566	.559
FC1	.485	.474	.467	.331	**.820**	.374	.418	.581	.444	.499	.541	.426
FC2	.470	.498	.466	.359	**.861**	.398	.411	.609	.425	.452	.544	.468
FC3	.462	.478	.395	.418	**.864**	.442	.508	.566	.465	.472	.528	.465
FC4	.535	.586	.444	.485	**.760**	.561	.529	.580	.563	.499	.604	.520
Trust1	.671	.640	.571	.545	.507	**.812**	.598	.626	.714	.628	.617	.611
Trust2	.591	.609	.598	.577	.468	**.833**	.589	.535	.614	.593	.575	.643
Trust3	.635	.614	.569	.563	.474	**.901**	.585	.538	.594	.546	.569	.649
Trust4	.670	.605	.511	.562	.479	**.879**	.551	.530	.612	.531	.581	.622
Trust5	.551	.515	.441	.537	.336	**.758**	.469	.375	.526	.457	.497	.572
Compatibility1	.572	.549	.636	.490	.538	.616	**.856**	.635	.599	.634	.560	.555
Compatibility2	.538	.535	.548	.565	.494	.556	**.916**	.606	.590	.643	.601	.588
Compatibility3	.543	.566	.573	.580	.469	.592	**.862**	.583	.618	.619	.598	.601
CO1	.491	.536	.562	.418	.655	.481	.600	**.865**	.548	.641	.544	.463
CO2	.594	.652	.637	.549	.650	.592	.683	**.908**	.631	.711	.585	.588
CO3	.538	.583	.528	.503	.604	.547	.592	**.891**	.576	.625	.565	.532
CO4	.520	.575	.445	.485	.563	.567	.533	**.826**	.532	.579	.494	.491
RA1	.535	.517	.531	.501	.517	.556	.605	.562	**.804**	.614	.567	.516
RA2	.558	.578	.585	.551	.492	.628	.604	.556	**.874**	.676	.625	.603
RA3	.626	.517	.533	.549	.506	.632	.577	.567	**.860**	.561	.513	.597
RA4	.626	.532	.443	.568	.405	.620	.485	.493	**.779**	.515	.510	.485
RD1	.544	.547	.555	.478	.513	.585	.646	.637	.648	**.901**	.567	.598
RD2	.497	.611	.538	.503	.564	.566	.642	.678	.637	**.894**	.561	.542
RD3	.545	.567	.470	.495	.466	.594	.613	.624	.606	**.848**	.534	.563
ITE1	.591	.608	.609	.561	.666	.608	.630	.622	.585	.600	**.899**	.704
ITE2	.569	.641	.606	.626	.616	.617	.618	.550	.617	.572	**.924**	.736
ITE3	.604	.590	.604	.652	.557	.625	.574	.540	.622	.543	**.903**	.748
EB1	.584	.589	.619	.558	.577	.577	.590	.552	.580	.586	.740	**.852**
EB2	.559	.603	.564	.608	.437	.644	.554	.466	.550	.572	.679	**.903**
EB3	.586	.617	.566	.610	.486	.662	.563	.519	.574	.546	.709	**.888**
EB4	.693	.645	.571	.651	.500	.698	.606	.540	.618	.550	.677	**.848**

Notes: Perceived consequences (PC); Facilitating conditions (FC); Complexity (CO); Relative advantages (RA); Results demonstrability (RD); Intention to engage (ITE); Engagement behaviour (EB).
