# Supplementary material for: A New Research Model for Artificial Intelligence–Based Well-Being Chatbot Engagement: Survey Study
Source: JMIR Hum Factors. 2024 Nov 11;11:e59908. doi: 10.2196/59908 (PMC11589509; doi:10.2196/59908)
Supplement: Multimedia Appendix 4 [file humanfactors_v11i1e59908_app4.pdf]

Multimedia Appendix 4 The values of Fornell-Larcker criterion

	Affect	Compatibility	Complexity	EB	FC	Habit	ITE	PC	RA	RD	Trust
Affect	**.944**										
Compatibility	.666	**.878**									
CO	.625	.691	**.873**								
EB	.664	.663	.595	**.873**							
FC	.537	.569	.709	.573	**.827**						
Habit	.619	.622	.561	.696	.488	**.807**					
ITE	.667	.668	.628	.803	.674	.675	**.908**				
PC	.584	.627	.614	.694	.594	.623	.647	**.861**			
RA	.633	.686	.656	.665	.579	.652	.669	.703	**.830**		
RD	.592	.719	.733	.645	.583	.558	.629	.601	.716	**.881**	
Trust	.645	.669	.626	.740	.544	.665	.679	.746	.733	.660	**.838**

Note: PC: Perceived consequences, FC: Facilitating conditions, CO: Complexity, RA: Relative advantages, RD: Results demonstrability, ITE: Intention to engage, EB: Engagement behaviour.

The square root of each construct's AVE should be bigger than its highest correlation with any other construct [1]. This criterion is met in our study.

Reference

1. Hair JF, Hult GT, Ringle C, Sarstedt M. A Primer on Partial Least Squares Structural Equation Modeling (PLS-SEM). Second. Sage. Thousand Oaks, CA: SAGE Publications Inc.; 2017. ISBN:9781483377445
